# Narrative review of diagnosis, management and treatment of dysphagia and sialorrhea in amyotrophic lateral sclerosis

**DOI:** 10.1007/s00415-024-12657-x

**Published:** 2024-08-29

**Authors:** Bogdan Bjelica, Susanne Petri

**Affiliations:** Department of Neurology, Hannover Medical School, 1, Carl-Neuberg-Strasse, 30625 Hannover, Germany

**Keywords:** ALS, Amyotrophic lateral sclerosis, Motor neuron disease, Dysphagia, Sialorrhea

## Abstract

The degenerative motor neuron disorder amyotrophic lateral sclerosis (ALS) frequently leads bulbar symptoms like dysarthria, dysphagia, and sialorrhea, in approximately one-third of cases being the initial symptom. Throughout the disease, more than two-thirds of ALS patients experience dysphagia, regardless of the region of onset. In this review, we aimed to offer an updated overview of dysphagia and sialorrhea in ALS, covering its diagnosis, monitoring, and treatment in clinical practice. Regular assessment of dysphagia and sialorrhea during each patient visit is essential and should be a standard aspect of ALS care. Early discussion of potential treatments such as high-calorie diets or percutaneous endoscopic gastrostomy (PEG) is crucial. Furthermore, this review highlights and discusses potential areas for improvement in both clinical practice and research.

## Introduction

Amyotrophic lateral sclerosis (ALS) is a heterogeneous neurodegenerative disorder marked by progressive degeneration of upper and lower motor neurons. Common clinical manifestations include progressive muscle wasting, weakness, dysarthria, dysphagia, and eventually, respiratory failure. Typically, the first symptoms appear in the limbs in about 65% of cases, while bulbar onset occurs in approximately 30% of cases, and respiratory onset is seen in about 5% [1]. ALS is considered a rare disease, with global incidence rates ranging from 0.5 to 3.6 cases per 100,000 people [2]. In the majority of cases, ALS occurs sporadically (sALS), likely due to a combination of genetic and environmental factors. However, in about 5–10% of cases, ALS is familial (fALS), with around 70% of these cases linked to genetic mutations in the *Superoxide dismutase 1* (*SOD1*) gene, *Chromosome 9 open reading frame 72* (*C9orf72*) gene, *Trans-activation response (TAR) DNA-binding protein 43* (*TARDBP*) gene, and *Fused in sarcoma* (*FUS*) gene [3].

Swallowing is a complex process that encompasses both volitional and reflexive activities, engaging over 30 nerves and muscles [4]. The swallowing process can be categorized into oral, pharyngeal, and esophageal stages based on the location of the bolus. Dysphagia, or difficulty swallowing, manifests through symptoms such as coughing and choking during or after a meal, difficulty in chewing, drooling, weight loss, and the risk of aspiration pneumonia. It can arise from a myriad of conditions, ranging from neurological disorders (such as stroke, Parkinson's disease, multiple sclerosis, myasthenia gravis, etc.) to psychiatric disorders and structural lesions [4]. Given that humans typically produce over half a liter of saliva daily and subconsciously engage in swallowing approximately every 1–3 min, the function of swallowing plays a crucial role in effective saliva management. In neuromuscular diseases like ALS, dysphagia and difficulty in the management of secretions (sialorrhea) are common symptoms [5]. It has been observed that in motor neuron diseases, the oral phase of swallowing is the most compromised, followed by the pharyngeal phase [6]. The importance of evaluating these symptoms is emphasized by the critical role the swallowing mechanism plays in safeguarding the airway, sharing significant anatomical features with it. Some serious medical complications such as dehydration and malnutrition can also arise due to dysphagia [4]. Furthermore, it is well-known that dysphagia increases the risk of death 7.7-fold in ALS patients [7]. The underlying reasons for dysphagia in ALS patients are nuclear or supranuclear lesions of the hypoglossus, vagus, and glossopharyngeal nerves. These result in atrophy and weakness of the tongue along with problems in closure of the soft palate and the larynx [8].

To the best of our knowledge, there is a lack of recent comprehensive reviews regarding dysphagia and sialorrhea in ALS. This review aims to present an updated summary of the knowledge surrounding dysphagia in ALS, offering insights into its diagnosis, monitoring, and treatment in everyday clinical practice.

### Frequency of dysphagia in ALS

As mentioned before, the most common initial symptom of ALS is progressive weakness in the muscles of the extremities. However, in one-third of patients, bulbar symptoms such as dysarthria, dysphagia, and sialorrhea are the first complaints [9]. The incidence of dysphagia in ALS patients differs according to the region of symptom onset. It is more frequently observed and tends to appear earlier in individuals with bulbar onset [10]. Throughout the course of disease, dysphagia is present in approximately 80% of the ALS patients, independent from the region of symptom onset [11]. Perry et al. conducted a retrospective cohort study using the Pooled Resource Open-Access ALS Clinical Trials (PRO-ACT) database, encompassing de-identified clinical patient records from 23 Phase II/III clinical trials and one longitudinal study conducted between 1990 and 2015 [12]. In their study, 6,149 ALS patients were included, responding to question 3 on the ALS Functional Rating Scale Score-Revised (ALSFRS-R), which addresses swallowing problems on a 5-point ordinal scale from 4 for "Normal" to 0 for "Nothing by mouth (NPO); exclusively parenteral or enteral feeding." Dysphagia was defined as a change in the self-reported score from 4 to < 4. The authors reported a cumulative incidence of new onset dysphagia, which was 44% at 1 year and 64% at 2 year post-trial enrollment for those with spinal onset, and 85% and 92% for those with bulbar onset [12].

Wang et al. reported a pooled prevalence of sialorrhea of 30.8% in their meta-analysis. Prevalence of mild, moderate, and severe sialorrhea was 25.1%, 11.2%, and 10.5%, respectively [13].

### Diagnosing and screening dysphagia in ALS

Diagnosing, assessing, and monitoring dysphagia in ALS patients present significant challenges owing to the absence of validated clinical tools and the considerable variation in methods across different medical centers. Currently, there is a lack of internationally accepted practice guidelines for evaluating bulbar function in ALS, and limited knowledge about the routine clinical use of assessment protocols [14]. The ALSFRS-R score and its bulbar subscale (covering only three items: swallowing, salivation and speech) is the main scale used in clinical practice nowadays [15]. Other noteworthy screening tools for early dysphagia risk identification in neurological disorders including ALS are: the Eating Assessment Tool-10 (EAT-10) [16], voluntary cough airflow [17], the bedside 3-oz water swallow test (WST) [18] and Sydney Swallow Questionnaire (SSQ) [19]. A recent validation study by Diamanti et al. assessed the DYALS (Dysphagia in Amyotrophic Lateral Sclerosis) questionnaire across 197 patients from 16 Italian ALS centers, concluding that the DYALS scale is reliable, manageable, and easily applicable for dysphagia screening in ALS [20]. Furthermore, Xia et al. conducted a monocentric study comparing the performance of ALSFRS-R bulbar subscale, WST, EAT-10, and SSQ in 68 ALS patients, using Videofluoroscopic Swallowing Study (VFSS) as the gold standard reference test. All four tests effectively identified patients with unsafe swallowing and aspiration, with EAT-10 demonstrating superior performance in diagnosing unsafe swallowing and aspiration [21]. These findings underscore the evolving landscape of dysphagia assessment tools in ALS and the importance of continued research in refining diagnostic approaches.

For those who show no dysphagia on screening, instrumental assessments such as VFSS and Fiberoptic Evaluation of Swallowing (FEES) should be implemented [22, 23]. These methods continue to serve as the gold standards for dysphagia assessment in neurological conditions, however they were found to be underutilized in multidisciplinary ALS clinics [14]. In the study by Briani and colleagues, VFSS findings were compared with FEES and pharyngo-oesophageal manometry findings in 23 patients with motor neuron disease (10 with amyotrophic lateral sclerosis, seven with progressive bulbar palsy, and six with progressive spinal muscular atrophy) [6]. VFSS demonstrated significantly greater sensitivity (92%) in detecting swallowing impairment. However, the specificity of VFSS was 0%, as all patients without dysphagia also exhibited radiological swallowing abnormalities. The authors concluded that VFSS could identify early abnormalities in patients without dysphagia who later developed the condition. Table 1 shows some of the instrumental and non-instrumental tools for diagnosing, screening and monitoring dysphagia in ALS patients.

**Table 1:** Tools for assessment of dysphagia in ALS

	Items/Scoring	Description
Self-reported measures		
ALSFRS-R score bulbar subscale [24]	3/total score: 12; higher scores indicating better self-reported function	Assessing swallowing, salivation and speech
EAT-10 [16]	10/total score: 40; higher scores indicating worse self-reported function	Each item presents a statement outlining a scenario that could pose a challenge for individuals experiencing difficulties with swallowing
SSQ [19]	17/total score: 1700; higher score indicating the greater risk of oropharyngeal dysphagia	Each question is scored using a visual analog scale (100 mm); subscales: global, physical and swallow related QoL; the only exception is the Q12 with numerical scoring scale from 0 to 5
DYALS [20]	10/total score: 10; higher score indicating more severe dysphagia	Newly validated in ALS; All items are dichotomous (yes/no); subscales: dysphagia for solids and dysphagia for liquids
Bed side tests		
3-oz WST [18]	–	Patients should drink approximately 30 ml of water from a cup within a 10-s timeframe while seated; patients with no coughing, interruption or wet-hoarse voice are considered normal
Voluntary cough airflow [17]	–	Measuring the airflow generated during a consciously initiated cough; the patient is instructed to take a deep breath and then voluntarily initiate a forceful cough
Instrumental assessments		
VFSS [25]	–	Gold standard; necessitates costly instrumentation and the expertise of highly trained personnel; minimal radiation exposure;
FEES [26]	–	Gold standard; experienced personnel and instrumentation needed; enables a direct examination of the laryngeal adductor reflex and offers the flexibility of being repeated as often as necessary, given its absence of radiation exposure

*ALSFRS-R* ALS Functional Rating Scale Score-Revised; *EAT-10* Eating Assessment Tool-10; *SSQ* Sydney Swallow Questionnaire; *DYALS* Dysphagia in Amyotrophic Lateral Sclerosis; *WST* Water Swallow Test; *VFSS* Videofluoroscopic Swallowing Study; *FEES* Fiberoptic Evaluation of Swallowing

### Treatment of dysphagia and sialorrhoea

Prioritizing proactive treatment for dysphagia and weight loss in all ALS patients is one of the most impactful objectives in their comprehensive care plan, since a 30% elevated risk of death accompanies each 5% weight loss in malnourished ALS patients, leading to an overall more than sevenfold increased risk of mortality [27–29]. The initial management of dysphagia involves several approaches, including dietary counseling, modification of food and fluid consistency (such as blending food and adding thickeners to liquids), prescription of high caloric diets, patient and caregiver education on feeding and swallowing techniques (such as supraglottic swallowing), and implementing postural changes. In addition, techniques like the “chin-tuck maneuver,” involving flexing the neck forward during swallowing, are employed to protect the airway [30].

According to the German guidelines, treatment should be offered when patients are experiencing suffering, weight loss, dehydration, and are at risk of aspiration. The first steps should involve nutritional counseling and prescribing high-caloric liquid nutrition, along with logopedic intervention [31]. In a prospective, double-blind, randomized, placebo-controlled clinical trial involving 212 ALS patients across 12 sites within the clinical and scientific network of German motor neuron disease centers, there no life-prolonging effect of a high-caloric fatty diet was observed in the overall ALS cohort. Nevertheless, post-hoc analysis unveiled a notable survival benefit within the subgroup of fast-progressing patients [32]. In addition, patients in the placebo group exhibited an increase in neurofilament serum levels, whereas those in the high-caloric fatty diet group demonstrated a decrease in neurofilament serum levels [33]. Finally, the most recent meta-analysis evaluating the efficacy, safety, and tolerability of a high-caloric diet in ALS patients suggested that high-caloric supplementation is generally safe and well-tolerated. However, it has not been demonstrated to be effective in improving weight and functional disability [34].

Enteral tube feeding represents a safe method to ensure adequate nutrition in patients with severe dysphagia. Various methods include nasogastric (NG) tubes, percutaneous endoscopic gastrostomy (PEG), PEG with jejunal extension (PEG-J), surgical gastrostomy, percutaneous endoscopic jejunostomy (PEJ), and surgical jejunostomy [35]. Another way is the percutaneous radiologic gastrostomy (PRG), which is less invasive [36]. These procedures can be performed on an outpatient or inpatient basis in settings such as the endoscopy suite, radiology suite, or operating room [35]. Fine-bore NG are suitable for short-term feeding, but they pose challenges such as easy displacement, high visibility, discomfort for the patient, and elevated risk of ulceration, aspiration pneumonia, and oro­pharyngeal secretions [37]. PEG with pre-intervention antibiotic treatment, gradual restoration of feeding, and peri-interventional support is recommended for cases of advanced dysphagia and weight loss [31]. In the most recent Cochrane review, results from 23 non-randomized studies comparing enteral tube feeding to oral feeding in individuals with ALS were examined. Notably, two prospective and five retrospective studies indicated an extended survival for individuals with ALS after undergoing PEG compared to those without PEG, even after accounting for potential confounding factors in multivariate analysis. However, four prospective, 10 retrospective, and two post hoc analysis studies did not demonstrate a survival advantage associated with PEG. All of these findings collectively supported the conclusion that there is no evidence indicating that enteral tube feeding enhances survival, nutritional status, or QoL in patients with ALS compared to the continuation of oral feeding alone [35].

Pharmacological treatment options for sialorrhea include pirenzepine, ipratropium bromide spray, scopolamine transdermal patch, amitriptyline, sublingual application of atropine 1% eye drops, and ultrasound-guided injection of incobotulinumtoxin A into the parotid and submandibular salivary glands [31]. Fractionated radiation of salivary glands (7–8 Gray) is considered as an option in cases of treatment-resistant sialorrhea [38]. The latest Cochrane review identified only four randomized controlled trials with 110 ALS patients. There was low-certainty or moderate-certainty evidence supporting the effectiveness of botulinum toxin B injections into salivary glands and moderate-certainty evidence for the use of oral combination of 20 mg dextromethorphan hydrobromide and 10 mg quinidine sulfate in treating sialorrhea in motor neuron disease. However, the evidence comparing radiotherapy to botulinum toxin A injections and scopolamine patches was too uncertain to draw conclusive conclusions [39].

Treatment of dysphagia and sialorrhoea requires specialists from several areas and is a good example for the need of multidisciplinary teams for the treatment of patients with ALS. It should include speech therapists, gastroenterologists and dieticians [30, 40].

## Future directions

As dysphagia assessments often involve patient self-reporting, and interventions may include dietary and/or postural restriction and/or modification, cognitive engagement of patients becomes crucial. Egan et al. observed that people with dementia develop dysphagia and mealtime difficulties as dementia progresses [41]. Regarding ALS, Francis et al. conducted a systematic review of the literature to explore how mild to moderate cognitive impairment associated with ALS impacts a patient's ability to understand and manage oropharyngeal swallowing function. They found that no study explicitly investigated this topic. In the future, research should explore how the presence of cognitive and behavioral impairments ranging from including mild cognitive impairment to clinical manifest frontotemporal dementia, influences the diagnosis and management of dysphagia in patients with ALS.

Furthermore, the optimal timing for offering PEG to ALS patients and whether it is more beneficial than a high-caloric diet in early stages of dysphagia and weight loss remains unclear, as there are no randomized controlled trials that have investigated enteral tube feeding versus a high-calorie diet, mainly due to ethical reasons. Certainly, this issue is not relevant to patients with severe dysphagia, who are unable to swallow and therefore require PEG placement for maintenance of enteral nutrition. There is a need for prospective cohort studies to assess and compare outcomes among patients with PEG placement at different levels of dysphagia and nutritional status. Considering the insufficient exploration of QoL associated with gastrostomy in ALS [35], it is crucial to delve deeper into this aspect in future studies, encompassing both patients and their caregivers.
